# Toward an understanding of the increase in enzymatic hydrolysis by mechanical refining

**DOI:** 10.1186/s13068-018-1289-3

**Published:** 2018-10-25

**Authors:** Tiago de Assis, Shixin Huang, Carlos Eduardo Driemeier, Bryon S. Donohoe, Chaehoon Kim, Seong H. Kim, Ronalds Gonzalez, Hasan Jameel, Sunkyu Park

**Affiliations:** 1 Department of Forest Biomaterials, College of Natural Reseources, NC State University, Raleigh, NC USA; 2 Department of Chemical Engineering, Pennsylvania State University, University Park, PA USA; 3 Brazilian Bioethanol Science and Technology Laboratory (CTBE), Brazilian Center for Research in Energy and Materials (CNPEM), Campinas, SP Brazil; 4 Biosciences Center, National Renewable Energy Laboratory, Golden, CO USA

**Keywords:** Sugarcane bagasse, Autohydrolysis pretreatment, Mechanical refining, Enzymatic hydrolysis, Fiber morphology, Fiber internal delamination, Cellulose crystallinity, Fiber porosity

## Abstract

**Background:**

Mechanical refining is a low-capital and well-established technology used in pulp and paper industry to improve fiber bonding for product strength. Refining can also be applied in a biorefinery context to overcome the recalcitrance of pretreated biomass by opening up the biomass structure and modifying substrate properties (e.g., morphology, particle size, porosity, crystallinity), which increases enzyme accessibility to substrate and improves carbohydrate conversion. Although several characterization methods have been used to identify the changes in substrate properties, there is no systematic approach to evaluate the extent of fiber cell wall disruption and what physical properties can explain the improvement in enzymatic digestibility when pretreated lignocellulosic biomass is mechanically refined. This is because the fiber cell wall is complex across multiple scales, including the molecular scale, nano- and meso-scale (microfibril), and microscale (tissue level). A combination of advanced characterization tools is used in this study to better understand the effect of mechanical refining on the meso-scale microfibril assembly and the relationship between those meso-scale modifications and enzymatic hydrolysis.

**Results:**

Enzymatic conversion of autohydrolysis sugarcane bagasse was improved from 69.6 to 77.2% (11% relative increase) after applying mechanical refining and an increase in enzymatic digestibility is observed with an increase in refining intensity. Based on a combination of advanced characterizations employed in this study, it was found that the refining action caused fiber size reduction, internal delamination, and increase in pores and swellability.

**Conclusions:**

A higher level of delamination and higher increase in porosity, analyzed by TEM and DSC, were clearly demonstrated, which explain the faster digestibility rate during the first 72 h of enzymatic hydrolysis for disc-refined samples when compared to the PFI-refined samples. In addition, an increased inter-fibrillar distance between cellulose microfibrils at the nano–meso-scale was also revealed by SFG analysis, while no evidence was found for a change in crystalline structure by XRD and solid-state NMR analysis.

## Background

Enzymatic hydrolysis of carbohydrates is one of the economic bottlenecks for the production of biofuels and biochemicals derived from monomeric sugars when lignocellulosic biomass is used as a feedstock. The high capital expense of reactors due to large reaction volumes and long reaction times (around 2–4 days), high costs of enzymes ($5.38 USD per kg protein) [1] and incomplete carbohydrate conversion (biomass recalcitrance) associated with enzymatic hydrolysis have a significant impact in the overall economics of biorefineries. Typically, enzymatic digestibility of biomass is directly affected by a combination of factors including substrate physical characteristics and chemical composition, chemistry and severity of pretreatment, type and dosage of enzymes, and other process conditions [2].

To overcome physical/structural and chemical/compositional barriers associated with lignocellulosic substrates, a pretreatment step can be used to reduce or remove the lignin barrier and disrupt the multiscale hierarchical lignocellulose architecture, improving enzyme accessibility for better cellulose saccharification. Pretreatment is an expensive unit operation and many factors must be evaluated during the selection of a low-cost and effective pretreatment process, including the following: production of highly digestible pretreated material, minimal sugar degradation or production of toxic compounds (carboxylic acids, furans, phenolic), low-cost reactors, minimal waste production, high sugar concentration, fermentation compatibility, lignin recovery, and low energy consumption [2]. Among various pretreatment options, autohydrolysis paired with mechanical refining has been suggested as a particularly cost-effective technology, where the absence of external catalysts creates the possibility to reduce both capital and operational costs, as well as significantly reduce environmental impacts compared to using a mineral catalyst-aided pretreatment [3–5].

Mechanical refining can be applied to any pretreated biomass to increase enzymatic digestibility and is agnostic to the specific chemical composition of the feedstock. Mechanical refining is a low-capital and well-established technology used by the pulp and paper industry to improve the bonding ability of fibers allowing the formation of a strong fiber network. Mechanical refining can also be used in a biorefinery context to overcome biomass recalcitrance. The mechanical refining action opens up biomass structure and modifies many substrate properties (e.g., increase of surface area, increase of pore size, reduction of particle size), which increases substrate accessibility and may result in the improvement of enzymatic digestibility [6]. Mechanical refining acts by at least three mechanisms that change fiber structure and morphology: cutting (fiber length reduction), shearing (fiber surface fibrillation), and compression (internal fiber crushing and delamination). The mechanisms occur simultaneously but at different relative levels depending on the refining technology [7]. Literature reports suggest that mechanical refining can be used to enhance sugar recovery using especially low enzyme dosages [8, 9] and to reduce pretreatment severity and enzyme loadings while still reaching same level of sugar recovery [4, 10].

Several characterization methods have been used to identify the altered substrate properties and measure the extent of their modification by mechanical refining, and determine the effect of those modifications on subsequent enzymatic hydrolysis. Light microscopy and scanning electron microscopy were used to access the effect of mechanical refining on fiber morphology. The analysis of microscope images showed that refining caused separation of cells, surface fibrillation, internal delamination and generation of fines. These changes collectively contributed to increased accessible surface area and enhance enzymatic hydrolysis [7, 11–13]. Fiber quality analyzer and laser diffraction were used to measure particle size of unrefined and refined biomass. The refining action, especially at high refining intensity, tended to reduce particle size and increase the abundance of fines due to fiber cutting and fibrillation. Reduction of particle size was usually correlated with an increase in enzyme-accessible surface area [7–15]. Water retention values, dye adsorption and differential scanning calorimetry were used to access the effect of mechanical refining on biomass surface area. Refining processes increased cell wall porosity and surface area, which contributed to increase the exposure of carbohydrates to cellulolytic enzymes [9, 10, 12, 13, 15, 16]. The effect of mechanical refining on cellulose crystallinity was also evaluated. A reduction of crystalline regions was observed via X-ray diffraction as a result of the compression and shear forces experienced by the fiber cell wall during mechanical refining. The reduction of crystallinity was believed to increase biomass digestibility [12, 17].

X-ray diffraction and other spectroscopic techniques (e.g., infrared, nuclear magnetic resonance) can only analyze structural changes at the molecular level. However, the structural and architectural changes that mechanical refining generates within the fiber cell wall and at the scale of microfibril interactions, and the relation between those meso-scale properties and enzymatic hydrolysis have not been fully explored. It is known that the structural hierarchy inside the fiber cell wall is complex across multiple scales, including the molecular scale (cellulose intra-molecular and inter-chain hydrogen bonding), nanoscale (assembly of cellulose chains into microfibrils), mesoscale (assembly of microfibrils in a matrix of polymers), and microscale (tissue level). Sum frequency generation vibrational spectroscopy is one alternative that can be used to detect the “crystalline” assembly of microfibrils without the interference of amorphous components [18]. In this work, we used sum frequency generation vibrational spectroscopy to evaluate the effect of mechanical refining on the meso-scale microfibril assembly and the relationship between those meso-scale modifications and enzymatic hydrolysis.

The objective of this work was to evaluate the extent of fiber cell wall disruption and what physical properties can explain the improvement in enzymatic digestibility when pretreated lignocellulosic biomass is mechanically refined. To address this objective, we performed a fundamental study to evaluate the modification of several substrate properties (particle size, swellability, pore area, crystallinity, chemical structure and morphology) when lab- and pilot-scale refining using PFI and disc refiners are applied to sugarcane bagasse after autohydrolysis pretreatment.

## Results

### Enzymatic hydrolysis

**Fig. 1:**
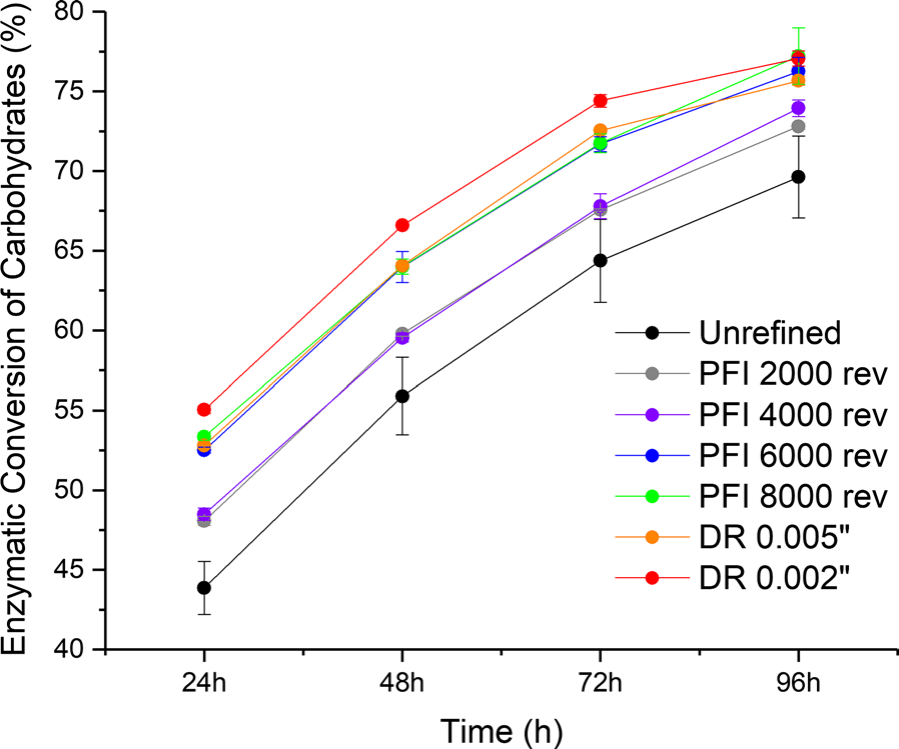
Carbohydrate conversion of unrefined and refined pretreated sugarcane bagasse samples after PFI and disc refining at different intensity levels after 24, 48, 72 and 96 h of enzymatic hydrolysis. Results are the average of duplicates

Figure 1 shows the enzymatic hydrolysis conversion for refined (PFI refiner and disc refiner) and unrefined samples. Mechanical refining improved enzymatic conversion of carbohydrates from 69.6 to 77.2% after 96 h (11% relative increase). For both refining methods, the increase in refining intensity improved carbohydrate conversion. The enzymatic hydrolysis profile for the refined samples using 2000 and 4000 PFI revolutions is similar, which showed the lowest improvement in carbohydrate conversion. Samples refined at 6000 and 8000 PFI revolutions and the disc-refined sample with a gap of 0.005 in. also presented similar enzymatic hydrolysis profiles with intermediate enhancement of carbohydrate conversion. The highest improvement was observed for the disc-refined sample with a gap of 0.002 in. Overall, disc-refined samples presented a faster carbohydrate conversion during the first 72 h of enzymatic hydrolysis when compared to the samples refined using a PFI refiner. After 72 h of enzymatic hydrolysis, the difference in carbohydrate conversion between PFI- and disc-refined samples was marginal.

### Macroscopic morphology

**Fig. 2:**
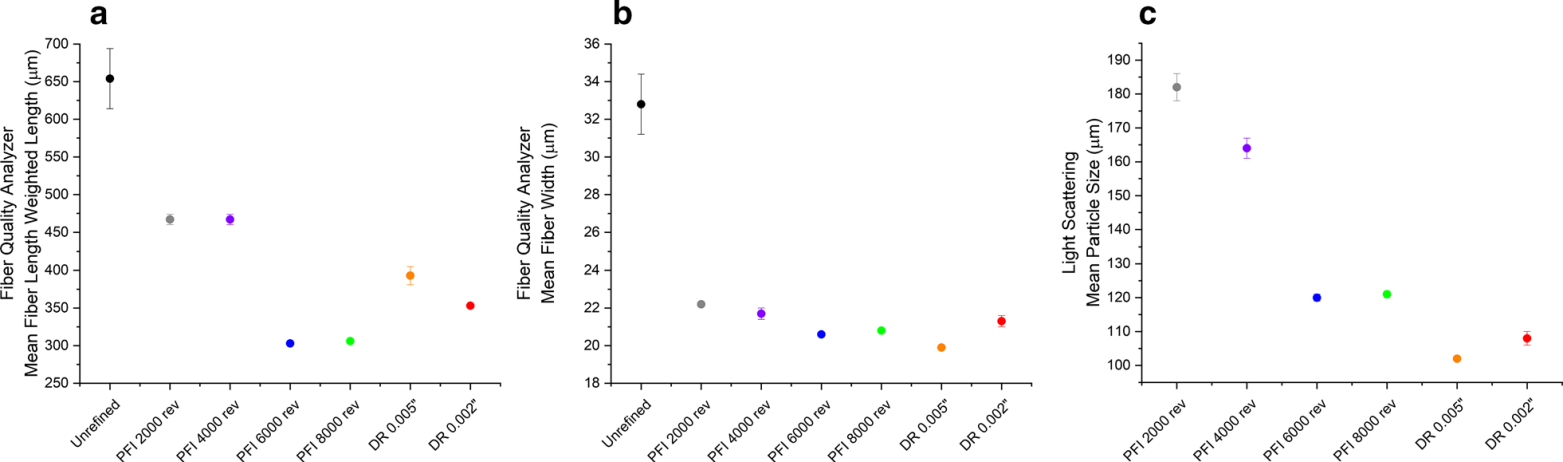
Mean fiber length-weighted length, mean fiber width and mean particle size of unrefined and refined pretreated sugarcane bagasse after PFI and disc refining at different intensity levels using fiber quality analyzer (FQA) and light scattering. **a** The mean fiber length-weighted length obtained from FQA. **b** The mean fiber width obtained from FQA. **c** The mean particle size obtained from light scattering. Results are the average of triplicates

Both refining methods decreased the length-weighted length of fibers bigger than 30 μm (Fig. 2a) and a general decrease of particle size distribution with the increase of refining intensity was also observed, confirming that both refining methods promote particle size reduction. The mean volume weighted particle size decreased from 180 to 120 μm when PFI refining intensity was increased from 2000 to 8000 revolutions (Fig. 2c). Similar reduction in particle size with the increase of refining intensity, when PFI and disc refining is applied in pretreated biomass, has been previously observed [7, 8, 10–12, 14]. Reduction in particle size is believed to improve enzymatic hydrolysis due to the associate increase in surface area.

A decrease in fiber width was observed when the unrefined sample is compared to the refined samples (Fig. 2b). This behavior can be explained based on the presence of non-uniform and coarsely fiberized particles (fiber bundles) in the unrefined sample. It is expected that the measured average width of a sample having fiber bundles would be bigger than the average width of single fibers. On the other hand, the refined samples show similar average width (~ 21 μm) suggesting that mechanical refining has caused cell–cell separation at middle lamella increasing the amount of single fibers relative to fiber bundles.

### Pore structure

**Fig. 3:**
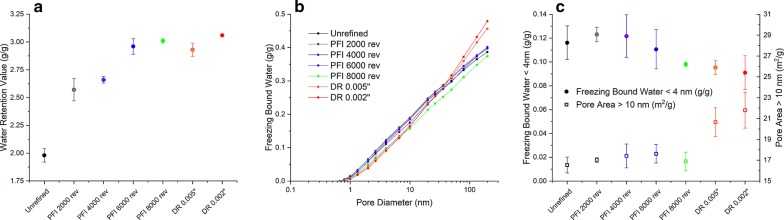
Water retention value (WRV) and thermoporometry profiles of unrefined and refined pretreated sugarcane bagasse after PFI and disc refining at different intensity levels. **a** The water retention value. **b** The cumulative distribution of freezing bound water as a function of pore diameter on a log scale with base of 10. **c** The porosity indexes in two different ranges of pore diameter derived from the freezing bound water (FBW) profile: FBW in pores < 4 nm and pore area associated with pores > 10 nm. Curves and results are the average of triplicates

Figure 3a clearly shows the increase of WRV with the increased refining intensity. The enhancement of swellability is more prominent between unrefined and refined samples. Among refined samples, swellability improves at a lower extent with the increased refining intensity. It is known that during water retention measurement of water-swollen fibers, most of the free or bulk water present within fiber lumens and in the spaces between adjacent fibers will be removed by centrifugation. The water remaining after centrifugation is mainly localized within the cell wall structure (inside pores, cavities or void spaces) and on the outer surfaces of fibers. Therefore, it can be concluded that mechanical refining is improving the water uptake of fibers within the cell wall and on the fiber surface. Many authors have reported a direct relationship between refining intensity and WRV for pretreated biomasses [9, 10, 12, 15]. A strong correlation between WRV and enzymatic hydrolysis is usually observed.

Figure 3b shows the FBW profiles for unrefined and refined samples. While PFI refining promoted only slight changes in the FBW profiles, disk refining promoted notable gains in FBW for pore diameters greater than ~ 10 nm. It is worthwhile to analyze FBW profiles looking separately into different length scales. Following a previous study [19], we separately analyzed FBW < 4 nm and the 10–200-nm range (Fig. 3c), the latter expressed as pore area in units of m^2^/g. In the < 4 nm range, FBW changes are small, with PFI ≥ 6 k and disk refining showing slight reduction of FBW compared to unrefined pretreated bagasse. This result is most likely due to enlarging of some pores as a result of mechanical action, which then do not contribute anymore to the < 4-nm range. For the 10–200-nm range, disk refining increased pore area and porosity in a magnitude not observed after PFI refining. The increase in cell wall porosity measured by DSC has also been reported somewhere else [16] and associated with the improvement in enzymatic hydrolysis.

### Crystalline structure—Molecular scale

**Fig. 4:**
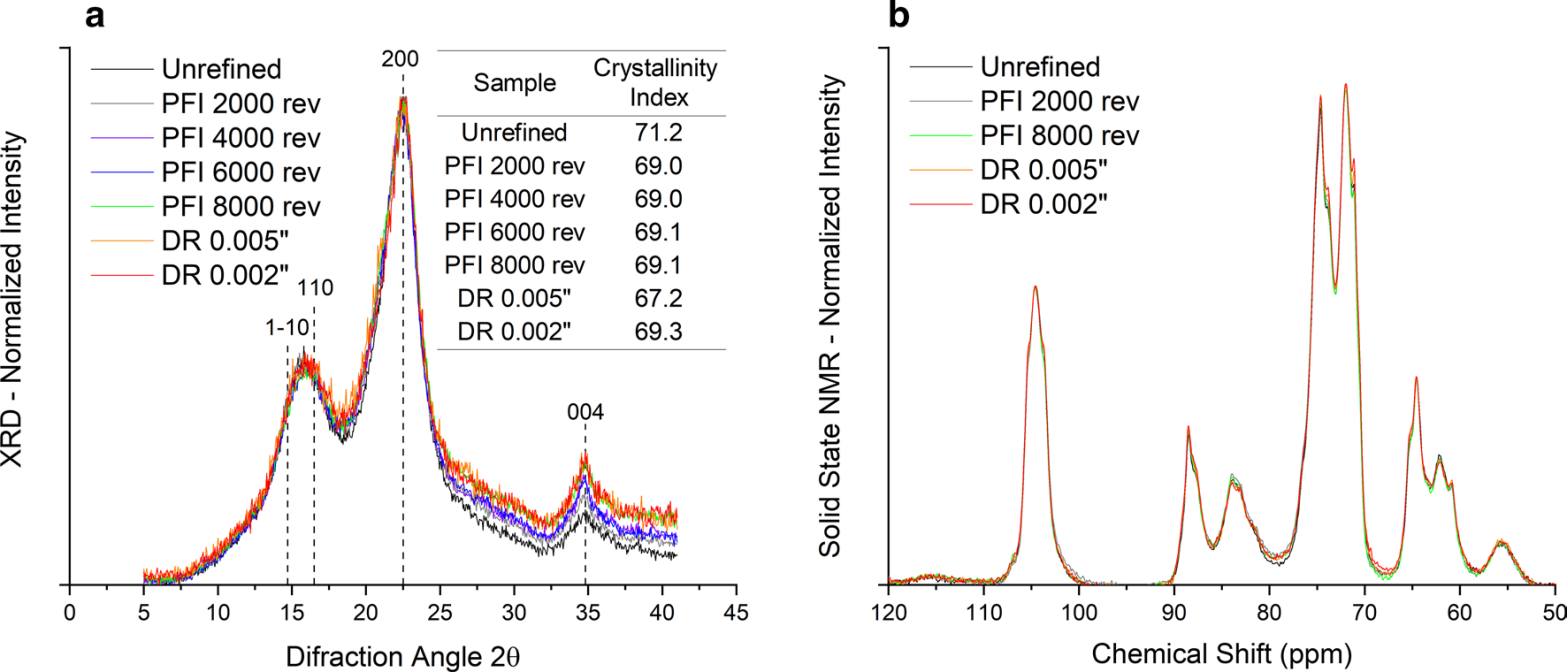
X-ray diffractograms (XRD) and solid-state nuclear magnetic resonance (NMR) spectra for unrefined and refined pretreated sugarcane bagasse after PFI and disc refining at different intensity levels. **a** The normalized XRD and calculated crystallinity index. **b** The normalized solid-state NMR spectra. XRD and NMR results are based on a single measurement

The X-ray powder diffractograms (Fig. 4a), normalized at the maximum intensity of (200) lattice diffraction (2*θ* around 22.5°) for unrefined and refined samples, do not show a substantial difference, and so are their CIs calculated according to the peak height method [20]. In addition, the solid-state NMR spectra also support the same observation (Fig. 4b). Noteworthy, thermochemical pretreatments cause the sharpening of cellulose diffraction peaks [21, 22] that has been attributed to cellulose co-crystallization, but such changes in diffraction peaks are not observed after refining. Hence, based on the presented results, there was no strong evidence of change in cellulose crystals induced by the mechanical forces of the refining processes. However, contrasting results were observed by other authors who reported a significant reduction in crystallinity due to the refining action [12, 17]. It was believed that the refining forces have caused distortion or destruction of crystalline domains and consequent reduction of crystallinity. Reduction of cellulose crystallinity is believed to improve enzymatic hydrolysis.

### Crystalline structure—Microfibrillar scale

SFG is a second-order non-linear optical process, which selectively detects the molecules or functional groups where centro-symmetry is broken. In plant cell walls, amorphous hemicellulose and lignin are negligible in the SFG analysis because they are randomly arranged. Only certain alkyl (C–H) and hydrogen-bonds (OH) vibration modes of crystalline cellulose can meet the non-centrosymmetric selection role and produce strong peaks in SFG spectra. For this reason, SFG can selectively probe crystalline cellulose without spectral interference from the matrix of polysaccharides [23]. In addition, the overall spectral shape and the relative CH/OH peak intensity/area ratio in SFG spectra are sensitive to the lateral packing of cellulose microfibrils in biomass [18, 24, 25].

**Fig. 5:**
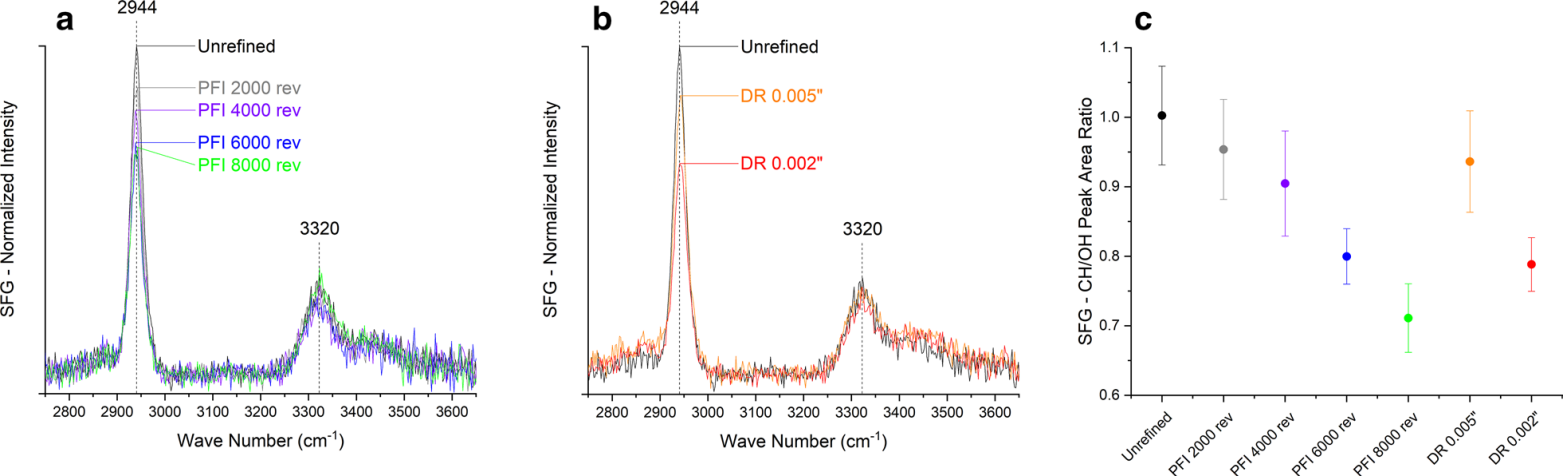
Sum frequency generation (SFG) spectra for unrefined and refined pretreated sugarcane bagasse after PFI and disc refining at different intensity levels. **a**, **b** The normalized SFG spectra for PFI- and disc-refined samples, respectively. **c** SFG CH/OH peak area ratio calculated from normalized SFG spectra. SFG results are the average of triplicates

Figure 5a and b shows the SFG spectra of unrefined and refined samples, which are also normalized by OH peak intensity at 3320 cm^−1^. Weakening in peak intensity at the 2944 cm^−1^, which is assigned to the asymmetric CH_2_ stretch vibration of exocyclic CH_2_OH groups of cellulose, is observed in PFI- and disc-refined samples when the refining intensity is increased. Peak area ratio of CH region (2750–3050 cm^−1^) over OH region (3150–3650 cm^−1^) of each SFG spectrum also decreases with more intense refining (Fig. 5c). Both CH/OH peak area ratios of the PFI-refined sample with refining intensity at 8000 revolutions (0.71 ± 0.05) and the disc-refined sample with gap size 0.002 in. (0.79 ± 0.04) are much smaller than the unrefined sample (1.00 ± 0.07). This decreasing trend in both the 2944 cm^−1^ peak intensity and the CH/OH peak area ratio should be attributed to changes in packing patterns of crystalline cellulose after refining since no significant variation in crystallinity has been shown in XRD before and after the refining (Fig. 4a). The SFG peak intensity change at 2944 cm^−1^ would indicate randomization in cellulose crystal packing after refining [26]. In addition, as a result of the lower SFG CH/OH peak area ratio presented by the refined samples, increased inter-fibrillar distances between cellulose microfibrils in nano–meso-scale are also suggested [18].

### Microscopic morphology

**Fig. 6:**
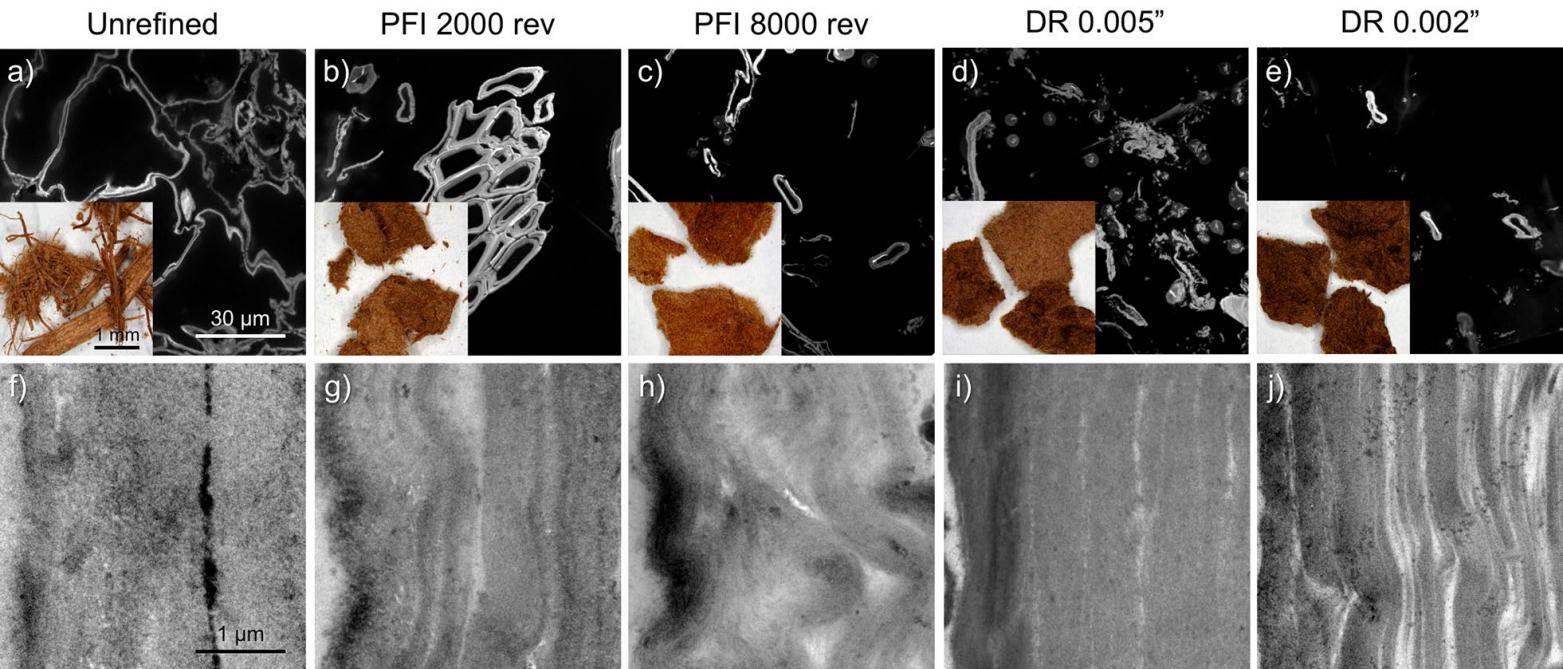
Morphology of unrefined and refined pretreated sugarcane bagasse after PFI and disc refining at different intensity levels using stereoscope microscopy (**a**–**e**, insets), confocal scanning laser microscopy (CSLM) (**a**–**e**) and transmission electron microscopy (TEM) (**f**–**j**)

At the particle scale, all samples in the stereoscope micrographs share a brownish color (Fig. 6a–e, insets). Both disc- and PFI-refined particles form clumps of finely fiberized biomass that appears uniform in particle size. On the other hand, the unrefined sample is coarsely fiberized, not clumped and non-uniform in particle size.

The CSLM microscopy (Fig. 6a–e) shows the impact of refining at the tissue scale. The refined samples display varying levels of cell–cell separation at the middle lamella relative to the more intact unrefined material.

The TEM micrographs (Fig. 6f–j) display the range of cell wall delamination generated by the refining process. While there is a range in the level of structural disruption and delamination in the cell walls for the refined samples, it generally correlates with the refining intensity. The PFI-refined cell walls show fine small gaps and appear to have a finer delamination that gives the walls a loose and wavy appearance (Fig. 6h). An additional feature seen in the PFI-refined samples is that the delaminated walls have also usually become wavy as if the layers have folded under the compressive forces of the refining. The disc-refined material on the other hand, especially after passing through a 0.002 in. gap, displays extensive cell wall delamination with relatively large displacement between the layers (Fig. 6j). This observed difference in cell wall delamination patterns between the two mechanical refining methods is indicative of the difference in the way the two mill designs impart compressing and shearing forces on the biomass. Different works have reported various levels of particle size reduction, surface fibrillation and cell wall delamination when PFI and disc refining is applied on pretreated biomass [7, 11]. Those morphological changes are believed to improve enzymatic hydrolysis due to the associated increase in surface area.

## Discussion

The combination of advanced techniques applied in this work was used to identify lignocellulosic fiber properties modified by the mechanical refining action and determine the effect of those modifications on the carbohydrate enzymatic hydrolysis of pretreated lignocellulosic biomass.

The enzymatic hydrolysis of lignocellulosic biomass is a biochemical process typically limited by the number of accessible substrate (carbohydrate) sites to the enzymes. During the initial hours of the enzymatic hydrolysis, there are a sufficient number of accessible substrate sites to accommodate the available enzymes. At longer time, the number of accessible substrate sites decreases as a result of the hydrolysis process. There is a point where a significant portion of the enzymes can no longer find substrate sites to hydrolyze. At this point, the enzymes will start to compete for the same site, the rate of enzymatic hydrolysis will decrease and additional enzyme dosage will not change the rate of enzymatic hydrolysis. In other words, for long enzymatic hydrolysis (typically longer than 12 h), the number of accessible substrate sites will eventually limit the enzymatic hydrolysis process. According to this “site limiting theory”, a different pretreatment (e.g., physical, chemical) is needed to maximize the number of accessible substrate sites and further improve enzymatic hydrolysis [27].

In the presented study, mechanical refining was used to increase the enzyme accessibility to the carbohydrates of sugarcane bagasse previously pretreated with autohydrolysis process. Our results show that there is a significant difference in carbohydrate conversion between the refined and unrefined samples, especially at the first 48 h. This behavior indicates that the mechanically refined samples have higher amount of accessible carbohydrate sites to accommodate a higher number of enzymes, which allows a faster enzymatic hydrolysis. After 72 h, the concentration of accessible carbohydrate sites per number of enzymes decreases, which reduces the enzymatic hydrolysis rate. After 96 h, the refined samples presented higher carbohydrates conversion indicating that refining action has exposed a number of carbohydrate sites that were before hidden in the lignocellulosic matrix. The reduction in biomass particle size (FQA and light scattering), increase in fiber swellability (WRV), increase in fiber porosity (DSC) and delamination of fiber cell wall (TEM) caused by the mechanical refining action are indications of increased surface area and enhanced enzyme accessibility to substrate, which ultimately promotes the observed improvement in carbohydrate enzymatic hydrolysis. In other words, cutting, shearing and compressing forces on the pretreated biomass due to mechanical refining open up the fiber cell wall structure and increase the number of available carbohydrate sites that were before hidden inside the hierarchical cell wall structure.

CSLM microscopy shows that the uniformity of particle size distribution and cell–cell separation at middle lamella caused by refining is in line with particle size reduction (length and width) observed with FQA and light scattering analysis. The delamination of the cell wall illustrated by TEM images can be used to explain the improvement in water uptake observed during WRV measurements. Additionally, the observations made by thermoporometry are in line with the contrasting cell wall disruption mechanisms observed by TEM images for PFI and disc refining. Specifically, FBW gains between 10 and 200 nm are compatible with the cell wall delamination (wider gaps) promoted by disc refining. The higher level of cell wall delamination and higher increase in porosity for pores bigger than 10 nm can be used to explain the faster digestibility rate during the first 72 h of enzymatic hydrolysis for disc-refined samples when compared to the PFI-refined samples. The finer delamination and disruption features of the refined samples observed from TEM images also agree with the increase of inter-fibrillar distances between cellulose microfibrils in nano–meso-scale observed with SFG analysis. However, no evidence was found for a significant change in crystalline structure at molecular level according to XRD and solid-state NMR analysis. The multi-scale changes in the biomass morphology seen as tissue-scale disruption and cell wall delimitation reveal mechanisms of increased substrate surface area and improved enzyme accessibility.

## Conclusions

Enzymatic digestibility of autohydrolysis sugarcane bagasse was improved from 69.6 to 77.2% with the mechanical refining treatment. Based on a combination of advanced characterizations employed in this study, it was found that refining action caused fiber size reduction, internal delamination, and increase in pores and swellability, which explain the fastest digestibility rate during the first 72 h of enzymatic hydrolysis for disc-refining samples when compared to the PFI samples. In addition, increased inter-fibrillar distances between cellulose microfibrils in nano–meso-scale are also suggested, while no clear evidence was found for the change in crystalline structure.

## Methods

### Biomass

Autohydrolysis pretreatment was performed at Brazilian Bioethanol Science and Technology Laboratory (CTBE) in a 350-L stainless steel batch reactor using 15 kg of raw bagasse at solid:liquid ratio of 1:10. The autohydrolysis pretreatment was performed at 190 °C during 10 min. After pretreatment, the liquid fraction was separated and the remaining solid fraction (pretreated bagasse) was washed with sufficient tap water to eliminate any soluble fractions until neutral pH was achieved. The washed solid in a wet state was stored in a cold room. Pretreated bagasse was characterized using a Laboratory Analytical Procedure published by National Renewable Energy Laboratory (NREL) [28] presenting the following composition (dry basis): glucan = 56.5 ± 0.3; xylan = 7.4 ± 0.2; total lignin = 28.1 ± 0.4; ash = 4.1 ± 0.2.

### Mechanical refining (PFI refining and disc refining)

Mechanical refining was applied on the pretreated bagasse using both a PFI and disc refiner at North Carolina State University (NCSU). The PFI refiner is a batch processing piece of equipment where biomass is beaten between a roll with bars and a smooth-walled housing, both rotating in the same direction but at different speeds. The refining action is achieved through the differential rotational action and the application of loading between the roll and housing for a specified number of revolutions [29]. Each batch of refining was performed with 30 g (dry basis) of pretreated biomass at 10% insoluble solids content. Four refining intensities (2000, 4000, 6000 and 8000 revolutions) were evaluated.

The pretreated biomass was also refined using a 12-in. continuous disc refiner. The disc refiner is composed of two vertical disks with serrated and contoured surfaces. One disk rotates, while the other remains stationary. The pretreated biomass at 20% insoluble solids content was fed between the disks where a centrifugal force pushes the fibers out toward the perimeter of the disks. The abrasion experienced by fibers cuts, softens, rubs, and disperses them. The space between the disks can be widened or shortened to modify the refining intensity. In this study, two refining intensities were evaluated (disc gap = 0.002 and 0.005 in.).

The chemical composition of refined samples was assumed to be the same as the pretreated biomass presented in section “Biomass”. The mechanical refiners used in this study are closed-type equipment, where all the biomass processed is collected at the end of refining. In other words, there is no mass loss during the refining process, and therefore, no difference is expected in the composition between the pretreated biomass and refined samples.

### Enzymatic hydrolysis

Enzymatic hydrolysis was performed at NCSU for all unrefined and refined samples in 50-mL tubes using 5 FPU/g (dry basis) of pretreated and washed substrate, 10% insoluble solids content, pH 4.8–5.0 and 50 °C. Novozymes Cellic CTec 2 supplemented with 1/9 Cellic HTec 2 was used as the enzyme cocktail. Sodium acetate buffer was used for pH control. The incubator (Fine PCR COMBI-D24) was maintained at 50 °C and 15 rpm. During enzymatic hydrolysis, samples were taken at 24, 48, 72 and 96 h. Each sample was centrifuged for 10 min at 4400 rpm using an Eppendorf Centrifuge 5702. The supernatant was used for sugar determination (glucose and xylose) and for calculation of carbohydrate conversion during enzymatic hydrolysis [30].

### Macroscopic morphology (fiber quality analyzer and light scattering)

The HiRes fiber quality analyzer (FQA) from OpTest Equipment Inc. uses circular polarized light to measure the length and width of particles. During the measurements, a very dilute fiber suspension is pumped to a flow cell, where an infrared light source is located. The polarized light passes through the flow cell. If the polarized light strikes a fiber, a phase shift will occur, which will allow the light to pass through a second polarizer and reach the camera located on the opposite side of the flow cell. Only highly organized structure, such as cellulosic fibers, is able to cause a phase shift in the polarized light. Therefore, FQA will not detect air bubbles, ink or scale. Samples were diluted (~ 1 mg/L) and dispersed before each analysis using a British disintegrator for 15,000 revolutions. Particles with sizes ranging from 0.03 mm to 10.0 mm were measured and 10,000 particles were analyzed for each FQA run. Fiber width was measured for particles with size bigger than 0.2 mm. Particle length was measured as the true contoured length and reported as the length-weighted length (*L*_w_).

$$\left( {L_{\text{w}} = \frac{{\mathop \sum \nolimits n_{i} L_{i}^{2} }}{{\mathop \sum \nolimits n_{i} L_{i} }}} \right),$$

where *n* is the number of fibers and *L* is the contour length.

Particle size distribution was evaluated by light scattering using a Beckman Coulter LS13320 instrument with Universal Liquid Module at CTBE. Particles with sizes ranging from 0.38 to 2000 µm were measured. Dilute aqueous suspensions of the refined particulates were injected into the instrument, passing through a window illuminated by 780 nm laser light. The scattered light is detected by 126 photodetectors distributed in scattering angle. The Fraunhofer optical model (which assumes spherical particles) encoded in the instrument software was employed to convert detected light intensities into particle size distributions. Unrefined pretreated bagasse could not be characterized by light scattering because particle lengths exceeded the instrument limit (2 mm).

### Pore structure (water retention value and calorimetric thermoporometry)

Water retention value (WRV) was performed at NCSU to estimate the swelling capacity of fibers by measuring the amount of the water retained in a wet and swollen sample after centrifugation. With the centrifugation and consequent elimination of excess water located in fiber lumens and in spaces between adjacent fibers, the remaining water will be mostly located on the outer surfaces of fibers and within the cell wall. WRV measurements were executed according to the TAPPI standard procedure [31]. A pulp suspension was placed into a filtering glass tube of medium porosity (22 mm diameter) to yield 1400 g/m^2^ (approximately 0.5233 OD g). The filtering glass tube was centrifuged for 30 min at 0.9 relative centrifugal force to gravity. After centrifugation, samples were oven dried at 105 °C. The weights of the wet centrifuged sample (*m*_wc_) and the oven-dried sample (*m*_od_) were measured to calculate WRV = (*m*_wc_ − *m*_od_)/*m*_od_.

Calorimetric thermoporometry using differential scanning calorimetry (DSC) was performed at CTBE to assess the pore area and porosity profile as a function of pore size. DSC experiments are able to differentiate three categories of water absorbed in biomass: non-freezing bound water (NFBW), freezing bound water (FBW), free water (FW) [31]. FW is bulk water and it is measured with ice melting at 0 °C. FBW is confined inside the biomass capillaries and it is measured with ice melting below 0 °C [32]. Water present in the capillaries has a depressed melting temperature due to the curved interfaces in cavities. This temperature has a relationship with the pore diameter, which allows the evaluation of pore size. NFBW is located at the first layers of water adjacent to the biomass surface. As the water movement is restricted by its association with the surface, NFBW does not freeze [32]. The main output from the technique is the FBW profile, given as cumulative distribution as a function of pore diameter in the range of 1–200 nm. Thermoporometry analysis was executed using a DSC TA Q200 with an auto sampler and RCS90 cooling unit, according to the procedure described previously [33] and recently updated [19].

### Crystalline structure (X-ray diffraction (XRD), solid-state nuclear magnetic resonance (NMR), sum frequency generation (SFG) spectroscopy)

X-ray diffraction was used at NCSU to estimate the crystallinity index (CI) of the samples. The wide angle diffraction data were acquired using a Rigaku SmartLab X-ray diffractometer (CuKα radiation). The diffraction angle of 2*θ* was measured from 5° to 41° with a step size of 0.05° and 5 s of exposure at each step. Crystallinity index was calculated, based on the peak height method [20, 34] as the ratio between the estimated intensity of the crystalline peak (*I*_200_ − *I*_am_) and the total intensity (*I*_200_). *I*_200_ is the maximum intensity of (200) lattice diffraction (2*θ* around 22.5°) and *I*_am_ is the intensity at the valley between (200) and (110)/(1–10) peaks (2*θ* around 18.4°), where intensity from amorphous components may have a notable contribution (in addition to the contribution from the tails of the adjacent peaks).

^13^C solid-state NMR measurements were carried out to evaluate crystalline structure of the samples using a Bruker Avance II 500 MHz with 4 mm MAS probe [35]. The instrument was operated at frequency of 125.76 MHz and spinning speed of 5 kHz. Signals were scanned 3000 times with pulse delay of 3 s and contact time of 2 ms.

The SFG experiment was performed at Pennsylvania State University using the broadband SFG spectroscopic system [36, 37]. The synchronized 800 nm and tunable infrared (2.5–10 µm) are needed to make the SFG process possible. The SFG intensity was normalized with the IR profile and each SFG spectrum was averaged from 4 different locations on each sample. The SFG spectra were collected from 2750 to 3650 cm^−1^.

### Microscopic morphology (stereomicroscopy, confocal scanning laser microscopy (CSLM), and transmission electron microscopy (TEM)

The microscope work was performed at NREL. For stereo microscopic analysis, whole pieces of refined and unrefined samples were examined without further processing. Images were captured on a Nikon SMZ1500 stereomicroscope and captured with a Nikon DS-Fi1 CCD camera operated by a Nikon Digital Sight system (Nikon Instruments, Melville, NY).

For CSLM and TEM analysis, samples were preserved for structural characterization using microwave processing as described previously [38]. Briefly, samples were fixed in 2.5% gluteraldehyde buffered in 0.1 M sodium cacodylate buffer (EMS, Hatfield, PS) under vacuum. The samples were dehydrated with ethanol and acetone. After dehydration, the samples were infiltrated with LR White resin (EMS, Hatfield, PA) at room temperature for several hours to overnight. The samples were transferred to flat-bottomed capsules and the resin polymerized by heating to 60 °C for 48 h. LR White embedded samples were sectioned to ~ 300 nm for CSLM or ~ 60 nm for TEM with a Diatome diamond knife on a Leica EM UTC ultramicrotome (Leica, Wetzlar, Germany).

For CSLM imaging, the semi-thin-sectioned samples were positioned on glass microscope slides and stained with 0.1% acriflavine. Images were captured using a 60 × 1.4 NA Plan Apo lenses on a Nikon C1 Plus microscope (Nikon, Tokyo, Japan), equipped with the Nikon C1 confocal system, excited using 488 nm from a tunable Argon laser operated via Nikon’s EZ-C1 software.

For TEM imaging, the ultra-thin sections were positioned on 0.5% Formvar-coated copper slot grids (SPI Supplies, West Chester, PA). Grids were post-stained for 3 min with 2% aqueous uranyl acetate and 3 min with 1% KMnO_4_ to selectively stain for lignins. Images were taken with a 4 mega-pixel Gatan UltraScan 1000 camera (Gatan, Pleasanton, CA) on a FEI Tecnai G2 20 Twin 200 kV LaB6 TEM (FEI, Hilsboro, OR).

## Abbreviations

CI: crystallinity index
CSLM: confocal scanning laser microscopy
CTBE: Brazilian Bioethanol Science and Technology Laboratory
DR: disc refiner
DSC: differential scanning calorimetry
FBW: freezing bound water
FQA: fiber quality analyzer
FW: free water
NCSU: North Carolina State University
NFBW: non-freezing bound water
NMR: nuclear magnetic resonance
NREL: National Renewable Energy Laboratory
PFI: PFI refiner
SFG: sum frequency generation spectroscopy
TEM: transmission electron microscopy
USD: United States of America Dollars
WRV: water retention value
XRD: X-ray diffraction

## References

[1] Humbird D, Davis R, Tao L, Kinchin C, Hsu D, Aden A (2011). Process design and economics for biochemical conversion of lignocellulosic biomass to ethanol—dilute-acid pretreatment and enzymatic hydrolysis of corn stover.

[2] Alvira P, Tomás-Pejó E, Ballesteros M, Negro MJ (2010). Pretreatment technologies for an efficient bioethanol production process based on enzymatic hydrolysis: a review. Bioresour Technol.

[3] Batalha LAR, Han Q, Jameel H, Chang H, Colodette JL, Gomes FJB (2015). Production of fermentable sugars from sugarcane bagasse by enzymatic hydrolysis after autohydrolysis and mechanical refining. Bioresour Technol.

[4] Ertas M, Han Q, Jameel H, Chang H (2014). Enzymatic hydrolysis of autohydrolyzed wheat straw followed by refining to produce fermentable sugars. Bioresour Technol.

[5] Han Q, Jin Y, Jameel H, Chang H-M, Phillips R, Park S (2015). Autohydrolysis pretreatment of waste wheat straw for cellulosic ethanol production in a co-located straw pulp mill. Appl Biochem Biotechnol.

[6] Park J, Jones B, Koo B, Chen X, Tucker M, Yu J-H (2016). Use of mechanical refining to improve the production of low-cost sugars from lignocellulosic biomass. Bioresour Technol.

[7] Chen X, Kuhn E, Wang W, Park S, Flanegan K, Trass O (2013). Comparison of different mechanical refining technologies on the enzymatic digestibility of low severity acid pretreated corn stover. Bioresour Technol.

[8] Chen X, Tao L, Shekiro J, Mohaghaghi A, Decker S, Wang W (2012). Improved ethanol yield and reduced Minimum Ethanol Selling Price (MESP) by modifying low severity dilute acid pretreatment with deacetylation and mechanical refining: 1) experimental. Biotechnol Biofuels.

[9] Jones BW, Venditti R, Park S, Jameel H, Koo B (2013). Enhancement in enzymatic hydrolysis by mechanical refining for pretreated hardwood lignocellulosics. Bioresour Technol.

[10] Jones BW, Venditti R, Park S, Jameel H (2014). Comparison of lab, pilot, and industrial scale low consistency mechanical refining for improvements in enzymatic digestibility of pretreated hardwood. Bioresour Technol.

[11] Chen X, Wang W, Ciesielski P, Trass O, Park S, Tao L (2016). Improving sugar yields and reducing enzyme loadings in the deacetylation and mechanical refining (DMR) process through multistage disk and szego refining and corresponding techno-economic analysis. ACS Sustain Chem Eng..

[12] Liu W, Wang B, Hou Q, Chen W, Wu M (2016). Effects of fibrillation on the wood fibers’ enzymatic hydrolysis enhanced by mechanical refining. Bioresour Technol.

[13] van der Zwan T, Hu J, Saddler JN (2017). Mechanistic insights into the liquefaction stage of enzyme-mediated biomass deconstruction. Biotechnol Bioeng.

[14] Chen X, Shekiro J, Pschorn T, Sabourin M, Tao L, Elander R (2014). A highly efficient dilute alkali deacetylation and mechanical (disc) refining process for the conversion of renewable biomass to lower cost sugars. Biotechnol Biofuels.

[15] Jones BW, Venditti R, Park S, Jameel H (2017). Optimization of pilot scale mechanical disk refining for improvements in enzymatic digestibility of pretreated hardwood lignocellulosics. BioResources.

[16] Koo BW, Treasure TH, Jameel H, Phillips RB, Chang HM, Park S (2011). Reduction of enzyme dosage by oxygen delignification and mechanical refining for enzymatic hydrolysis of green liquor-pretreated hardwood. Appl Biochem Biotechnol.

[17] Zhang Y, Mu X, Wang H, Li B, Peng H (2014). Combined deacetylation and PFI refining pretreatment of corn cob for the improvement of a two-stage enzymatic hydrolysis. J Agric Food Chem.

[18] Makarem M, Sawada D, O’Neill HM, Lee CM, Kafle K, Park YB (2017). Dependence of sum frequency generation (SFG) spectral features on the mesoscale arrangement of SFG-active crystalline domains interspersed in SFG-inactive matrix: a case study with cellulose in uniaxially aligned control samples and alkali-treated secondary cell walls of plants. J Phys Chem C.

[19] Driemeier C, Oliveira MM, Curvelo AAS (2016). Lignin contributions to the nanoscale porosity of raw and treated lignocelluloses as observed by calorimetric thermoporometry. Ind Crops Prod.

[20] Segal L, Creely JJ, Martin AE, Conrad CM (1959). An empirical method for estimating the degree of crystallinity of native cellulose using the X-ray diffractometer. Text Res J.

[21] Driemeier C, Mendes FM, Santucci BS, Pimenta MTB (2015). Cellulose co-crystallization and related phenomena occurring in hydrothermal treatment of sugarcane bagasse. Cellulose.

[22] Langan P, Petridis L, O’Neill HM, Pingali SV, Foston M, Nishiyama Y (2014). Common processes drive the thermochemical pretreatment of lignocellulosic biomass. Green Chem.

[23] Barnette AL, Bradley LC, Veres BD, Schreiner EP, Park YB, Park J (2011). Selective detection of crystalline cellulose in plant cell walls with sum-frequency-generation (SFG) vibration spectroscopy. Biomacromol.

[24] Lee CM, Kafle K, Park YB, Kim SH (2014). Probing crystal structure and mesoscale assembly of cellulose microfibrils in plant cell walls, tunicate tests, and bacterial films using vibrational sum frequency generation (SFG) spectroscopy. Phys Chem Chem Phys.

[25] Park YB, Lee CM, Koo B-W, Park S, Cosgrove DJ, Kim SH (2013). Monitoring meso-scale ordering of cellulose in intact plant cell walls using sum frequency generation spectroscopy. Plant Physiol.

[26] Wang W, Chen X, Donohoe BS, Ciesielski PN, Katahira R, Kuhn EM (2014). Effect of mechanical disruption on the effectiveness of three reactors used for dilute acid pretreatment of corn stover part 2: morphological and structural substrate analysis. Biotechnol Biofuels.

[27] Banerjee G, Car S, Scott-Craig JS, Borrusch MS, Bongers M, Walton JD (2010). Synthetic multi-component enzyme mixtures for deconstruction of lignocellulosic biomass. Bioresour Technol.

[28] Sluiter A, Hames B, Ruiz R, Scarlata C, Sluiter J, Templeton D (2012). Determination of structural carbohydrate and lignin in biomass.

[29] TAPPI. Laboratory beating of pulp (PFI Mill Method)—T248 sp-00. 2000.

[30] Sluiter A, Hames B, Ruiz R, Scarlata C, Sluiter J, Templeton D (2006). Determination of sugars, byproducts and degradation products in liquid fraction process samples.

[31] TAPPI. Water retention value (WRV)—UM 256. 2011.

[32] Park S, Venditti RA, Jameel H, Pawlak JJ (2006). Changes in pore size distribution during the drying of cellulose fibers as measured by differential scanning calorimetry. Carbohydr Polym.

[33] Driemeier C, Mendes FM, Oliveira MM (2012). Dynamic vapor sorption and thermoporometry to probe water in celluloses. Cellulose.

[34] Park S, Baker JO, Himmel ME, Parilla PA, Johnson DK (2010). Cellulose crystallinity index: measurement techniques and their impact on interpreting cellulase performance. Biotechnol Biofuels.

[35] Park S, Johnson DK, Ishizawa CI, Parilla PA, Davis MF (2009). Measuring the crystallinity index of cellulose by solid state 13C nuclear magnetic resonance. Cellulose.

[36] Kafle K, Lee CM, Shin H, Zoppe J, Johnson DK, Kim SH (2015). Effects of delignification on crystalline cellulose in lignocellulose biomass characterized by vibrational sum frequency generation spectroscopy and X-ray diffraction. Bioenergy Res..

[37] Lee CM, Kafle K, Huang S, Kim SH (2016). Multimodal broadband vibrational sum frequency generation (MM-BB-V-SFG) spectrometer and microscope. J Phys Chem.

[38] Donohoe BS, Decker SR, Tucker MP, Himmel ME, Vinzant TB (2008). Visualizing lignin coalescence and migration through maize cell walls following thermochemical pretreatment. Biotechnol Bioeng.

